# In Vivo 3D Imaging of Retinal Neovascularization Using Multimodal Photoacoustic Microscopy and Optical Coherence Tomography Imaging

**DOI:** 10.3390/jimaging4120150

**Published:** 2018-12-12

**Authors:** Van Phuc Nguyen, Yanxiu Li, Michael Aaberg, Wei Zhang, Xueding Wang, Yannis M. Paulus

**Affiliations:** 1 Department of Ophthalmology and Visual Sciences, University of Michigan, Ann Arbor, MI 48105, USA; 2 Department of Biomedical Engineering, University of Michigan, Ann Arbor, MI 48105, USA; 3 Department of Radiology, University of Michigan, Ann Arbor, MI 48105, USA

**Keywords:** photoacoustic microscopy, optical coherence tomography, multimodal imaging, retinal neovascularization, vascular endothelial growth factor, PAM, OCT, VEGF

## Abstract

The pathological process of neovascularization of the retina plays a critical role in causing vision loss in several diseases, including diabetes, retinal vein occlusion, and sickle cell disease. Retinal neovascularization can lead to vitreous hemorrhage and retinal detachment, yet the pathological process of neovascularization is a complex phenomenon under active investigation. Understanding and monitoring retinal neovascularization is critically important in clinical ophthalmology. This study describes a novel multimodal ocular imaging system which combines photoacoustic microscopy (PAM) and a spectral domain optical coherence tomography (SD-OCT) to improve the visualization of retinal neovascularization (RNV), their depth, and the surrounding anatomy in living rabbits. RNV was induced in New Zealand rabbits by intravitreal injection of vascular endothelial growth factor (VEGF). The retinal vasculature before and after injection at various times was monitored and evaluated using multimodal imaging including color fundus photography, fluorescein angiography (FA), OCT, and PAM. In vivo experiments demonstrate that PAM imaging distinctly characterized the location as well as the morphology of individual RNV with high contrast at a safe laser energy of 80 nJ. SD-OCT was used to identify a cross-sectional structure of RNV. In addition, dynamic changes in the retinal morphology and retinal neovascularization were observed at day 4, 5, 6, 7, 9, 11, 14, 28, and day 35 after VEGF injection. PAM demonstrated high-resolution optical absorption of hemoglobin and vascular imaging of the retina and choroid with increased depth of penetration. With the current multimodal imaging system, RNV can be easily visualized in both 2D and 3D angiography. This multimodal ocular imaging system provides improved characterization of the microvasculature in a safe manner in larger rabbit eyes.

## 1. Introduction

Retinal neovascularization (RNV) is major causes of vision loss and blindness in the United State and around the world [1–3]. RNV develops when pathologic new blood vessels form in the retina. These RNV vessels lack the normal vascular morphology and can leak fluid, rupture, cause bleeding or scar tissue formation, or cause retinal detachment, leading to vision loss [4,5]. Neovascularization commonly appears at the optic nerve head and the major vascular arcades of the retina since they arise from the pre-existing retinal vasculature [3]. Neovascularization occurs in numerous retinal diseases, including diabetic retinopathy, retinopathy of prematurity, retinal vein occlusion, and sickle cell disease. RNV can be treated using several treatment methods, including panretinal photocoagulation, cryotherapy, anti-vascular endothelial growth factor (anti-VEGF), and vitrectomy. However, without treatment, vitreous hemorrhage and tractional retinal detachments can develop. Thus, early detection and accurate diagnosis of neovascularization is imperative to optimize visual outcomes. Currently available noninvasive imaging of retinal disease is based on imaging anatomical changes in the retina, including intraretinal and subretinal edema, hemorrhage, microaneurysms, and retinal pigment epithelium (RPE) changes.

The conventional imaging modalities used to monitor and evaluate retinal microvasculature and RNV include fluorescein angiography (FA), indocyanine green angiography (ICGA), scanning laser ophthalmoscopy (SLO), optical coherence tomography (OCT), and optical coherence tomography angiography (OCTA) [6–9]. Each imaging modality has its own strengths and limitations in assessing retinal diseases. For example, FA is commonly used for ocular vascular imaging. However, FA only visualizes the superficial capillary plexus [6]. ICGA is used to detect neovascular membranes beneath the retinal pigment epithelium (RPE) and through hemorrhages. However, both ICGA and FA cannot specify the depth of the vasculature, are invasive requiring the injection of an intravenous exogenous dye, and can cause nausea and vomiting in up to 10% of patients and in extreme cases can cause anaphylaxis and death [10]. OCT is now widely used in ophthalmology clinics and can provide high resolution, volumetric, non-invasive visualization of superficial retinal vascular disease and ocular structures by encoding the intensity variance resulting from blood perfusion. However, OCT and OCTA have difficulty visualizing the choroid and choriocapillaris vasculature, and OCT provides limited information on these important layers outside of thickness measurements [11]. OCTA provides both vascular and structural information, which permits assessment of vasculature with depth information; however, this method does not demonstrate leakage, and it provides limited visualization of microaneurysms. Furthermore, bulk motion between b-scans may produce line artifacts in the enface projection images, it is unable to visualize the choroid and choriocapillaris vasculature, and it has a restricted field of view often with artifacts [12,13]. PAM has a greater depth of penetration than other optical imaging techniques, such as OCT, due to the fact that light must only pass through the sample once to be absorbed by the tissue, whereas OCT evaluates that light that returns to the detector which thus has to pass through the sample twice and thus reduces the depth of penetration. SLO can achieve visualization of the smallest retinal capillaries, but SLO has limited image contrast and depth resolution, resulting in the inability to differentiate between vascular beds [14,15]. The best visualization of the retinal microvasculature requires the administration of exogenous contrast agents, which can have significant side effects and can limit its clinical utility. The development of an advanced noninvasive ophthalmological imaging technique to visualize ocular microvasculature and to understand RNV pathogenesis would be greatly beneficial to eye doctors.

Recently, photoacoustic microscopy (PAM) has been developed as an emerging, non-ionizing, and non-invasive imaging technique to evaluate various types of ocular tissues and to visualize vasculature in three-dimensions (3D) [16–19]. Compared to other imaging modalities, PAM can image ocular vasculature based on the inherent optical absorption of hemoglobin with high-resolution, high sensitivity, high-contrast, and high depth of penetration [20–28]. Therefore, this technique can provide label-free images without the assistance of exogenous contrast agent, resulting in no toxicity and no side effects [18,20,29,30]. PAM not only provides structural information, but it also provides functional information of the ocular vasculature. This unique strength of PAM could play an important role in accurately assisting of ophthalmic diagnosis by providing detailed morphological information of the vascular network [29,30]. PAM imaging can be divided into two groups: acoustic-resolution PAM (AR-PAM) and optical-resolution PAM (OR-PAM) [16,18,31]. For AR-PAM, a diffusing laser light is used to illuminate the tissues and the induced PA signals are detected by a focused ultrasound transducer. The focus beam of ultrasound transducer determines the lateral resolution of AR-PAM, whereas the axial resolution is determined by the ultrasound frequency and bandwidth [32–35]. In OR-PAM, both the excitation light and ultrasound detection are tightly focused onto the ocular tissue and co-registered in the confocal plane. The optical focal beam used to generate the PA signal determines the lateral resolution. However, the axial resolution is still determined by the ultrasound transducer parameters [36]. In this study, we will use the term PAM for OR-PAM, for simplicity purposes. Joen and Kelly Goss et al. have described the potential application of optical resolution photoacoustic microscopy (OR-PAM) to assess the progression of corneal neovascularization in mice [16,28,37]. By performing PAM at multiple optical wavelengths, their studies could provide the vessel oxygenation (SO_2_), which is important information for various ocular diseases [16,37]. Liu, Jiao, and Song et al. have introduced an integration of PAM and OCT to better visualize retinal vessels, choroidal vessels, and the RPE [22,31,38]. Recently, Dai et al. have developed an integrated PAM and OCT to monitor and evaluate laser-induced choroidal neovascularization in mice [39]. Previously, our group successfully used photoacoustic microscopy PAM and OCT to evaluate retinal and choroidal blood vessels in living rabbits with high temporal and spatial resolution. Furthermore, our group demonstrated that the integration of PAM and OCT provided multimodal imaging of both optical absorption and back-scattering of the retina [29,30]. Our custom-built multimodal-imaging platform performs imaging with high lateral spatial resolution of 4.1 µm for PAM and 3.8 µm for OCT at the focal plane of the objective with a high depth of penetration that allows visualization of both retinal and choroidal vasculature. Thus, PAM and OCT could be used to better characterize retinal neovascularization.

The aim of this study is to investigate a multimodal imaging system for monitoring retinal neovascularization using PAM, OCT, FA, and color fundus photography. Retinal neovascularization was generated using intravitreal injection of VEGF. Dual-modality PAM and OCT were performed in conjunction to monitor and evaluate the changes in the retinal morphology and the spatial extent of retinal neovascular changes.

## 2. Materials and Methods

### 2.1. Chemical Materials

Vascular endothelial grow factor (VEGF-165) was purchased from Shenandoah (Shenandoah Biotechnology Inc., PA, USA). Phosphate-buffered saline (PBS) was purchased from Gibco BRL, Life Technologies (Grand Island, NY, USA). Fluorescein (10%) was purchased from Akorn (Akorn, Lake Forest, IL, USA). Ketamine was ordered from the University of Michigan Pharmacy from JHP Pharmaceuticals (JHP Pharmaceuticals, Rochester, MI, USA). Xylazine was acquired from MWI Animal Health (Anased® LA, Boise, ID, USA). All reagents were used as received without further purification.

### 2.2. Animal Model Preparation

All animal experimental procedures were conducted in accordance with the ARVO (The Association for Research in Vision and Ophthalmology) Statement on the Use of Laboratory Animals in Ophthalmic and Vision Research, after approval by the Institutional Animal Care and Use Committee (IACUC) of the University of Michigan (Protocol number: PRO00006486, PI: Y. Paulus). Eight New Zealand rabbits (weighing 2.2–2.8 kg, 2–3 months of age, both genders) were used in this study. The rabbits were obtained from the Center for Advanced Models and Translational Sciences and Therapeutics (CAMTrasST) at the University of Michigan Medical School. Retinal neovascularization (RNV) was induced in the rabbit eye by injecting VEGF. Each rabbit model was initially anesthetized by intramuscular (IM) injection of ketamine (40 mg/kg IM, 100 mg/mL) and xylazine (5 mg/kg IM, 100 mg/mL). Anesthesia was maintained with a vaporized isoflurane anesthetic (1 L/min oxygen and 0.75% isoflurane) (Surgivet, MN, USA) during the RNV model preparation and in vivo imaging experiments. Then, the rabbit was placed on animal custom-built platform with a water-circulating blanket (TP-700, Stryker Corporation, Kalamazoo, MI, USA) to maintain the animal body temperature. VEGF injection was performed on left eyes. The right eyes were used as control. To generate RNV, the rabbits were injected of 100 µL VEGF-165 (10 µg/mL) dispersed in phosphate-buffered saline (PBS) (N = 8). The animal mucous membrane color, temperature, heart rate, and respiratory rate were monitored and recorded using a pulse oximeter (V8400D Capnograph & SpO2 Digital Pulse Oximetry, Smiths Medical, MN, USA) prior to the experiments. Tropicamide 1% ophthalmic and phenylephrine hydrochloride 2.5% ophthalmic were used to dilate the pupils of the rabbit. Topical tetracaine 0.5% was instilled in the eye for topical anesthesia, and lubricant (Systane, Alcon Inc., TX, USA) was used every minute during the experiment to prevent corneal dehydration. The rabbits were imaged with color fundus photography, FA, OCT, and PAM on days 0 (pre-injection), 4, 5, 6, 7, 11, 14, 28, and 35 after VEGF injection to observe the development of new vessels, as well as the morphology changed of the major blood vessel after RNV.

### 2.3. Color Fundus

To monitor the dynamic change of retinal vessels, all vessels were monitored before and after VEGF injections using 50-degree color fundus photography (Topcon 50EX, Topcon Corporation, Tokyo, Japan). Additionally, the color fundus images were used to measure the change of vessel size before and after RNV. To monitor the effect of the VEGF on adjacent vessels, color fundus images were acquired at different positions of the eye such as the optic nerve, the temporal medullary ray, the nasal medullary ray, the superior retina above the optic disc, and the inferior retina below the optic disc.

### 2.4. Fluorescein Angiography (FA)

After color fundus imaging, FA was employed to evaluate the vasculature network and confirm the development of new microvasculature. FA was performed using the Topcon 50EX as described by previous studies [30]. 0.2 mL sodium fluorescein solution (10%) (Akorn, Lake Forest, IL, USA) was injected in the marginal ear vein, and the images were captured immediately. Late-phase fluorescence photos were acquired at every minute for a period of at least 15 minutes.

### 2.5. Dual Optical-Resolution Photoacoustic Microscopy (OR-PAM) and Spectral Domain Optical Coherence Tomography (SD-OCT) Imaging System

**Figure 1:**
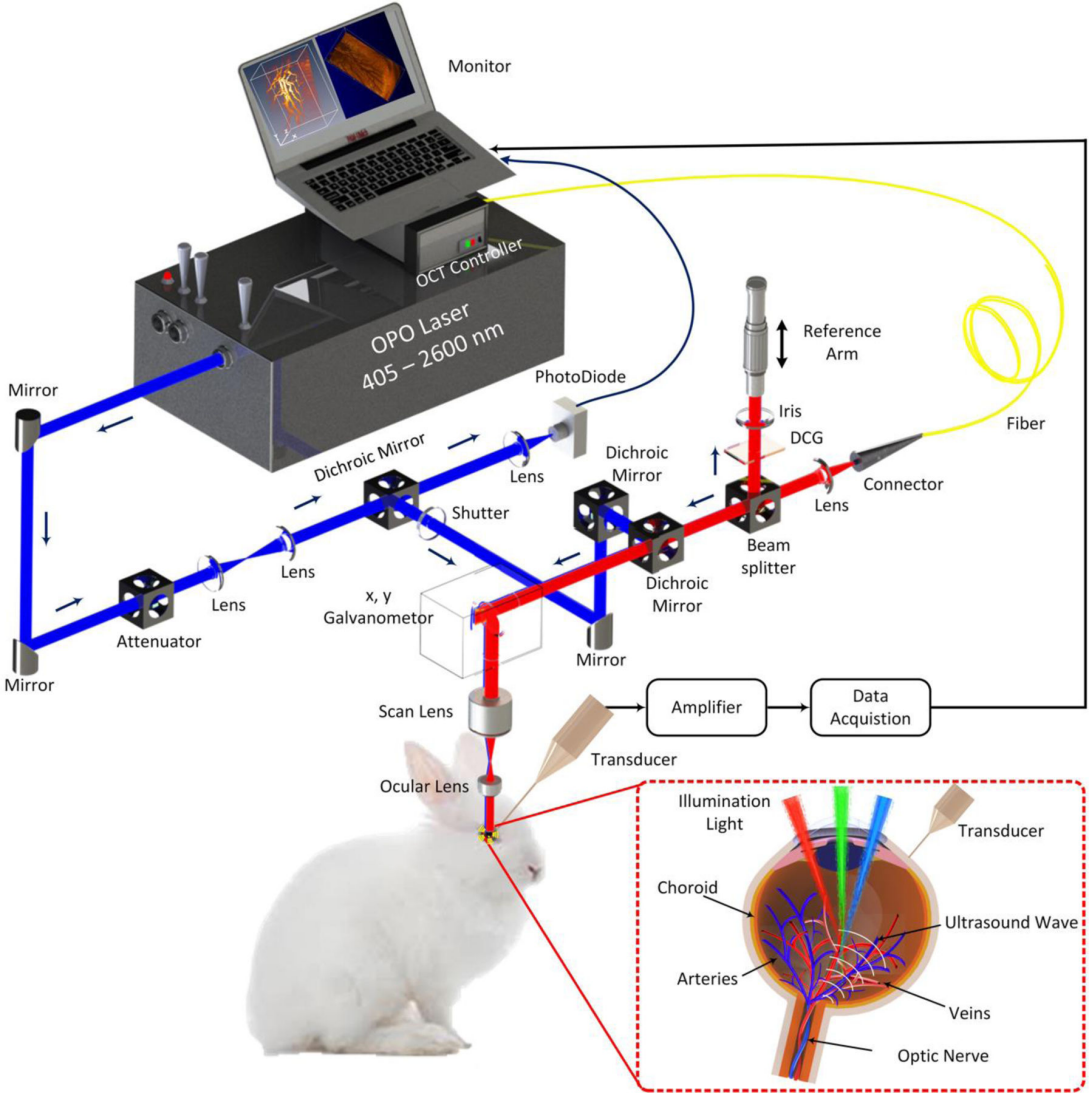
Schematic diagram of integrated optical-resolution photoacoustic microscopy (OR-PAM) and spectral domain optical coherence tomography (SD-OCT). Please note that the red, green, and blue light paths indicate that different wavelengths can be used to image the retinal blood vessels by using a tunable laser system. DCG is dispersion compensation glass.

To image the rabbit retinal vessels, a custom-built dual PAM and OCT system (Figure 1) was utilized to obtain all the imaging in this manuscript as previously described [30]. In brief, the photoacoustic imaging system is consisted of: (1) a nanosecond diode-pumped solid-state pulse laser used to generate short laser pulses (a pulse width = 3~5 ns and a pulse repetition rate = 1 kHz) and used as the illumination source, (2) a custom-built 27.0 MHz needle-shaped ultrasound transducer (Optosonic Inc., Arcadia, CA, USA) used to detect the laser-induced acoustic signals, (3) an optical two-dimensional galvanometer scanning head, (4) a telescope consisting of a scan lens (focal length 36 mm, OCT-LK3-BB, Thorlabs, Inc., Newton, NJ, USA) and an ophthalmic lens (OL, focal length 10 mm, AC080–010-B-ML, Thorlabs), (5) a high-speed digitizer at a sampling rate of 200 MS/s (PX1500–4, Signatec Inc., Newport Beach, CA, USA). The generated laser light was collimated, and delivered to a scanning head and raster-scanned by a two-dimensional galvanometer, which is a shared component with the spectral domain (SD)-OCT system. The scanned beam traveled through a telescope and was focused on the fundus by the rabbit eye optics. The PAM images were carried out at an optical wavelength of 580 nm, which matches the optical absorption peak of oxyhemoglobin. The laser light energy on the eye used to acquire images was less than 80 nJ at 580 nm, which is half of the American National Standards Institute (ANSI) safety limit [30]. The transducer was mounted in contact with the conjunctiva and aligned to enable accurate co-registering with the illuminating laser light. The transducer yields lateral and axial resolutions of 4.1 and 37.0 µm, respectively. 2D depth-sensitive PAM images were acquired by implementing horizontal scanning lines along the x-axis. For 2D image reconstruction, each sample was scanned along x- and y-directions using an optical-scanning galvanometer with a resolution of 6.1 *×* 6.1 µm2, while scanning depth (z-direction) was fixed at the focal depth of the imaging transducer. For a 1.5 *×* 1.5 mm2 field of view, the acquisition time was approximately 60 s.

To determine the maximum permissible exposure (MPE) for ocular imaging, the current study applied the American National Standards Institute (ANSI) safety limit standard (ANSI Z136.1–2007) [30,40,41]. The ANSI MPE limits for retinal exposure to nanosecond pulses in the 400 to 700 nm spectral range is 5.0 10*−*7 J cm*−*2 [30,40,41]. For a single-pulse MPE calculation, assuming a beam diameter matched to a fully dilated pupil (5 mm): Gaussian 1/e2 diameter: D = 5 mm (contains 61% of total pulse energy).

Fluence on the cornea:

(1) $$\Gamma =\frac{\text{E}}{\text{A}}=\frac{{0.61E}_{\text{p}}}{\text{π}{\left(\frac{\text{D}}{\text{2}}\right)}^{\text{2}}}$$

(2) $$\Gamma =3.11\;{\text{E}}_{\text{p}}\;\text{J}\cdot {\text{cm}}^{-2}\;(\text{where}\;\text{Ep}\;\text{is}\;\text{energy}\;\text{in}\;\text{joule})$$

(3) $$\Gamma <\text{MPE}=5.010^{-7}\text{J}\cdot {\text{cm}}^{\text{-2}}\;\text{for}\;\text{nanosecond}\;\text{visible}\;\text{light}$$

Thus, the max energy for single pulse exposure is

(4) $$\text{E}=\frac{\Gamma }{3.11}=160\;\text{nJ}$$

**Figure A1:**
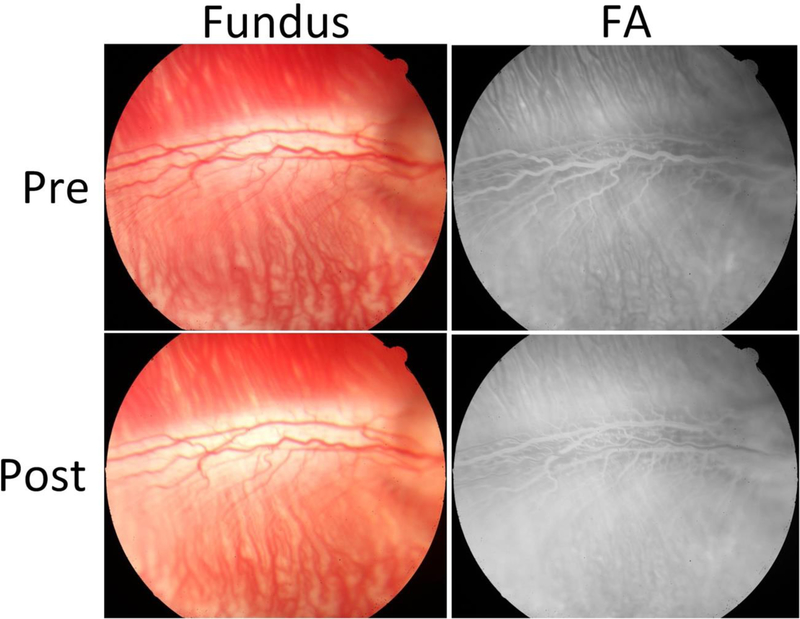
Color fundus photography of the retina before and after dual PAM and OCT experiment. As shown in this figure, the morphology of retinal vessels and retina is normal, indicating that the application of laser light at 80 nJ does not result in visible retinal damage.

Additionally, to visualize the margin of the blood vessel, 3D image reconstruction was performed using Amira software (FEI, Hillsboro, OR, USA). Furthermore, post-processing was performed on the 3D image to improve visualization of the blood vessel margins. Median filter function was applied to reduce background noise and unwanted signal surrounding the region of interest (ROI) occurred during the acquired PA signal, resulting in enhancement the quality of the PAM image. After 3D image reconstruction, the image segmentation was semi-automatically performed to classify the positions of retinal vasculature and neovascularization by applying segmentation function in Amira. In addition, the 3D structure of retinal neovascularization was also reconstructed to visualize the structure and estimate the detected diameter of blood vessels. To confirm that the applied laser energy used for in vivo imaging did not induce any severe effect to the retinal vessels, the rabbit was imaged with color fundus photography, and fluorescein angiography after acquiring PAM images (Figure A1). Immunohistochemistry was also performed to evaluate for evidence of inflammation, cell injury, and cell death as a sign of retinal neural death.

Spectral domain Optical Coherence Tomography (SD-OCT) was developed from a commercial SD-OCT system (Ganymede-II-HR, Thorlabs, Newton, NJ) by adding the ocular lens after the scan lens and a dispersion compensation glass in the reference arm [30]. A combination of two super luminescent diodes with center wavelengths of 846 nm and 932nm was used to excite the tissue. The lateral and axial resolutions were 3.8 µm and 4.0 µm, respectively. The OCT light source was coaxially aligned with the PAM system. The OCT image was acquired using two protocols: a preview OCT protocol and an average protocol. Both preview and average protocols used the A-line acquisition rate of 36 kHz. First, the review protocol was utilized to identify the position of selected areas and review the image quality. The cross-sectional B-scan image contained 512 A-lines. When the initial alignment is completed, average signal function was selected (average = 20) and high-resolution OCT images were captured, where 512 *×* 1024 A-lines were recorded per image with an image acquisition time of 0.103 s.

### 2.6. In Vivo PAM and OCT for Detection of Retinal Neovascularization

Retinal neovascularization (RNV) models were imaged by using dual optical-resolution photoacoustic microscopy (OR-PAM) and SD-OCT. The retinal vessels pre- and post-VEGF injection were visualized using the OR-PAM and SD-OCT imaging modalities. The rabbits were positioned on the imaging platform after anesthetic injection. The head and body of the rabbit were placed on two high-performance, custom-made stabilization platforms to minimize breathing and other motion artifacts. The regions of interest (ROI) were first imaged with color fundus photography and OCT. After acquiring OCT images, the PAM user control system was turned on, and the ROI was selected from the fundus camera integrated in the system. Then, a high-sensitivity needle ultrasonic transducer was mounted in the eye chamber, allowing it to move freely in 3D while not applying physical pressure on the rabbit eyes. The rabbit retinas were imaged with PAM, OCT, fundus photography, and FA at days 0 (baseline), 4, 5, 6, 7, 9, 11, 14, 28, and 35 after VEGF injection to monitor the pathologies process (i.e., presence of retinal neovascularization, size of retinal vessels, and stability of RNV). After the in vivo experiments, the rabbits were placed in a recovery area with a heat blanket, and vitals such as mucous membrane color, rectal temperature, heart rate, and respiratory rate were observed and documented until the rabbit was fully recovered. Then, the rabbit was returned to the animal facility and the body weight was monitored every day for 7 days.

### 2.7. Histological Analysis

All the rabbits were sacrificed thirty-five days after VEGF injection to evaluate the change in retinal vessels. Rabbits were euthanized by injection of intravenous injection of pentobarbital (euthanasia solution, 0.22 mg/kg I.V, 50 mg/mL) (Beuthanasia-D Special, Intervet Inc., Madison, NJ, USA). The eyeball and select tissues were collected aseptically from the euthanized rabbits. The isolated tissues were cut and fixed in neutral buffered formalin (10%) (VWR, Radnor, PA, USA) for a minimum of 48 h. The isolated eyeball was fixed in Davidson’s fixative solution (Electron Microscope Sciences, PA, USA) for 24 h to avoid retinal detachment. Then, the samples were transferred to an alcohol solution (50%) (Fisher Scientific, PA, USA) for an additional 24 h. Finally, the sample was transferred to a 70% alcohol solution and kept at room temperature for 24 h prior to embedding in paraffin. The fixed tissues were cut into 5 mm cross-sectional sections and embedded in paraffin. Subsequently, the tissues embedded in paraffin were sectioned into a thickness of 4 µm and stained with hematoxylin and eosin (H&E) for standard histopathological examination using a Leica autostainer XL (Leica Biosystems, Nussloch, Germany) under standard conditions. Images of the slides were captured using a Leica DM600 light microscope (Leica Biosystems, Nussloch, Germany) to detect the position of retinal neovascularization. Digital images were achieved with the BF450C camera. In addition, the retinal thickness was also quantitatively measured using LAS X software (Leica Biosystems, Nussloch, Germany).

### 2.8. Quantification of Retinal Neovascularization

To quantify the retinal neovascularization, we calculated the new vessel density and vessel diameter before and after VEGF at various times from day 0 to day 35 using Image J software (National Institute of the Health, Bethesda, MD, USA). To minimize the measurement error, the changes of vessel size were measured from eight different regions in the PAM, OCT and color fundus images. Images from each time point were manually registered to subsequent time points to correctly assess changes in RNV.

### 2.9. Statistical Analysis

All experiments were carried out a minimum of four times, and the final data points are presented as the mean *±* standard deviation (SD). Student’s t-tests were performed to compare two experimental conditions using the Microsoft Excel 2013 (Microsoft Corporation, Redmond, WA, USA), and *p ≤* 0.05 was considered as statistically significant (as shown in Dataset).

## 3. Results

**Figure 2:**
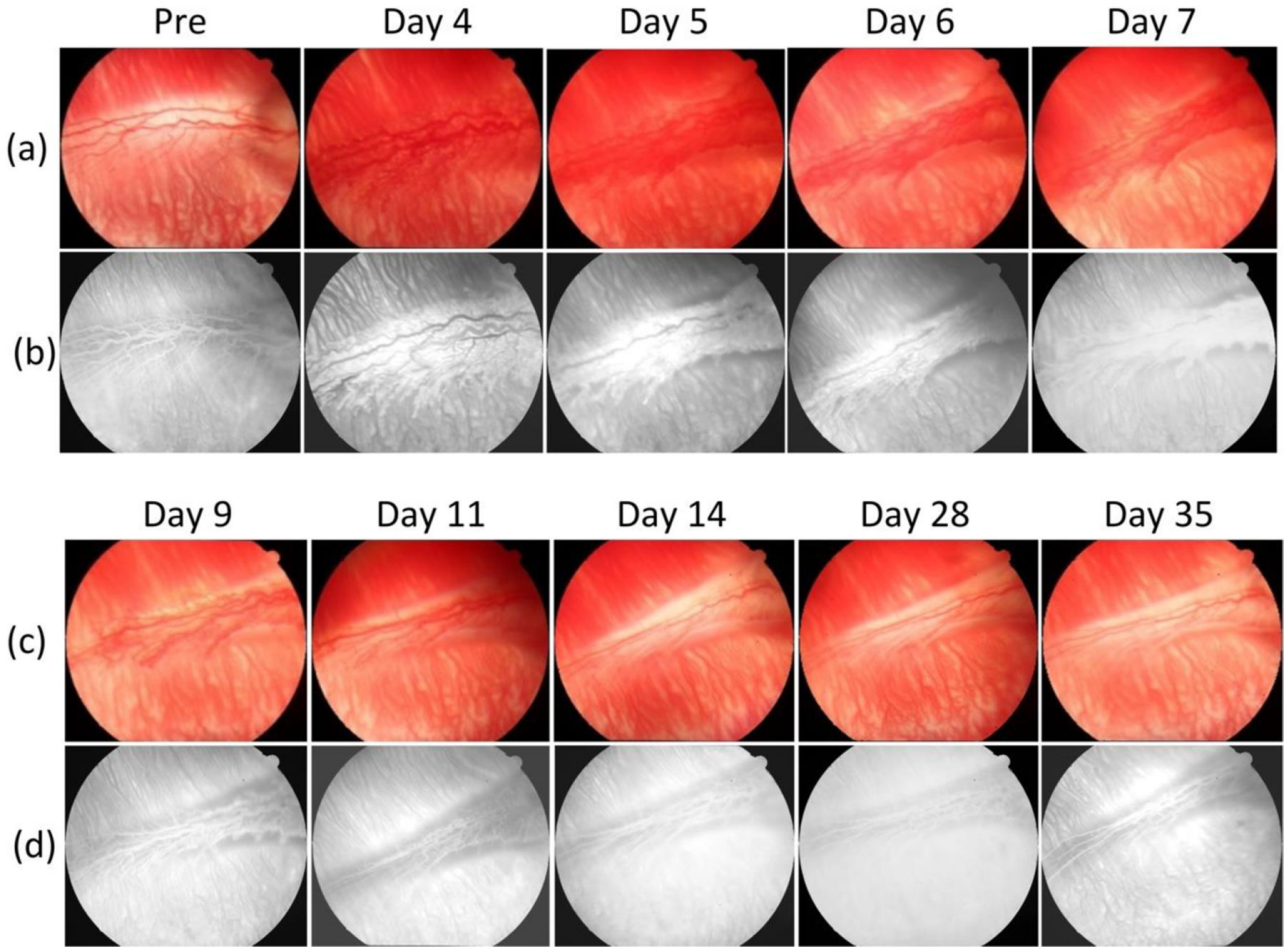
Color fundus photography imaging of retinal blood vessels in rabbits. (**a,c**) Color fundus photography of the retina acquired before and after retinal neovascularization (RNV) model at various times (days 4, 5, 6, 7, 9, 11, 14, 28, and 35). The fundus images show the morphology of retinal and choroidal vessels. (**b,d**) Fluorescein angiography images show retinal and choroidal vessels.

**Figure A2:**
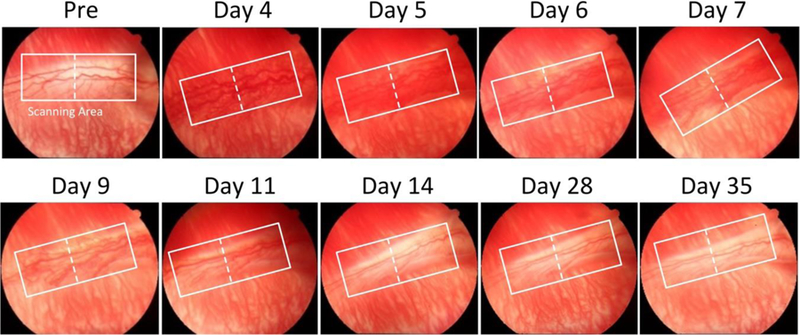
Color fundus photography of the retina before and after VEGF injection. The white rectangles indicate the selected scanning regions for PAM whereas the white dotted lines represent the selected scanning regions for OCT.

To evaluate the changes in retinal vessels after VEGF injection, the region of interest (ROI) was imaged, including the RNV and choroidal vessels with a field of view of 3 *×* 3 mm as shown in Figure A2. The PAM images of the retinal neovascularization were implemented at a wavelength of 580 nm due to strong optical absorption of oxyhemoglobin at that wavelength. Before PAM and OCT, the targeted retinal vessels and the scanning ROI were selected from color fundus camera and fluorescein angiography (FA). Figure 2 shows serial photograph images of major retinal vessels before and after VEGF injection at various times from day 0 to day 35 post-injection. Figure 2a depicts color fundus photographs of the retina before in vivo OCT and PAM imaging. The color fundus photograph demonstrates the morphology of the retinal vessels (white arrows), and choroidal vessels (white dotted arrows). Figure 2b shows FA images obtained after acquiring color fundus photography to co-register these images. The morphology of retinal and choroidal microvasculature is clearly visualized. FA images were used to evaluate the position of the developed neovascularization after RNV development. Please note that the retinal neovascularization initially developed at day 4 and peaked at day 5 to day 6. Then, the RNV vessels gradually decreased from day 7 to day 11 and turn to normal at day 14 to day 35. In comparison with the color fundus and FA images obtained before VEGF injection, the morphology of retinal vessels was significantly changed.

**Figure 3:**
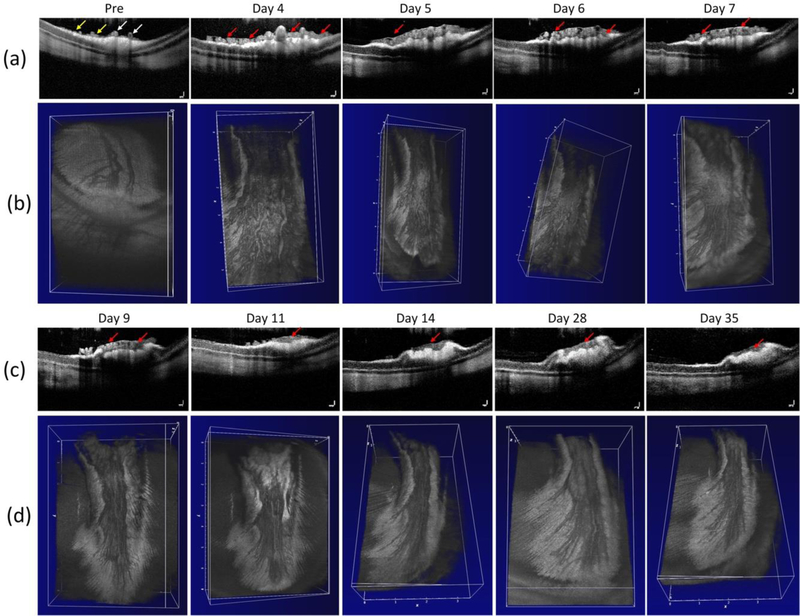
In vivo evaluation of a retinal neovascularization. (**a,c**) Cross-sectional B-scan OCT images acquired along the scanning lines from Figure A1. The OCT image showing choroidal vessels (CVs), retinal vessels (RVs), retinal neovascularizarion (RNV) and retinal layers. Yellow arrows show the position of the existing retinal blood vessels. Red arrows depict the location of RNV. (**c,d**) 3D volumetric OCT images for the retinal neovascularization.

Figure 3 exhibits the cross-sectional B-scan OCT images acquired along the dotted lines shown on the color fundus image in Figure A1. These OCT images were performed at different times before and after the RNV model and reveal the formation of new blood vessels. These OCT images present the change in the retinal layers. The yellow arrows show the position of the major retinal vessels whereas the red arrows indicate the position of RNV. Similar to color fundus and FA images shown in Figure 2, these OCT images demonstrate that the RNV was significantly increased at days 4, 5, 6, and 7. Then the RNV gradually decreased from day 9 to day 35. In addition, nerve fiber layers were obviously enlarged in comparison to control images. In addition, no retinal detachment was observed on the OCT image. This demonstrates that OCT can potentially visualize the formation of RNV. Figure 3c,d represents 3D OCT volumetric images of the retinal neovascularization. The OCT volumetric images clearly display a branching network of retinal neovascularization.

**Figure 4:**
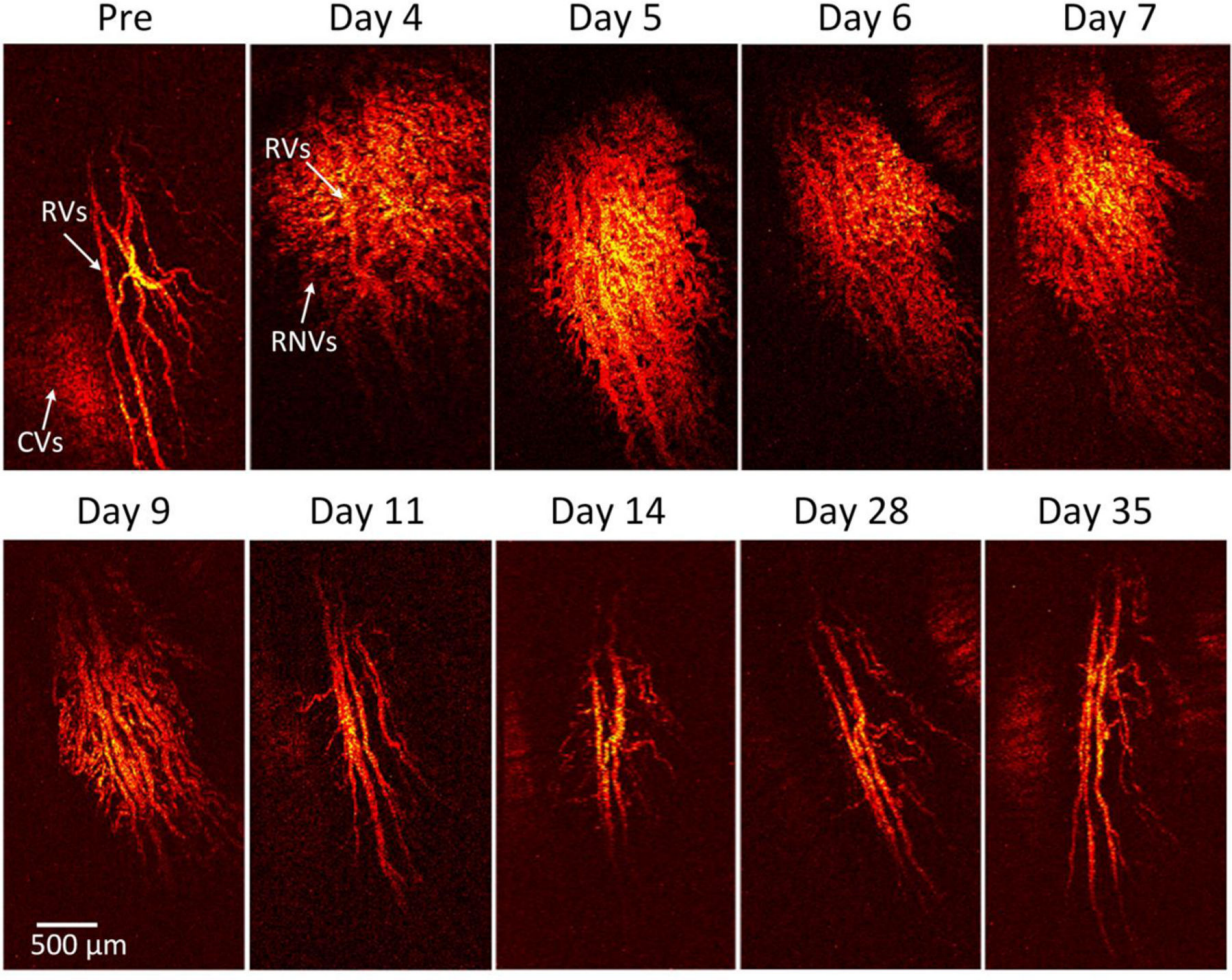
In vivo optical resolution (OR-PAM) images of retinal neovascularization. Maximum intensity projection (MIP) PAM images of the rabbit retinal vessels were acquired before and after retinal neovascularizarion (RNV) model at various times. The MIP OR-PAM images show clearly the structure of individual retinal blood vessels including choroidal vessels (CVs), retinal vessels (RVs), RNV and capillaries.

**Figure 5:**
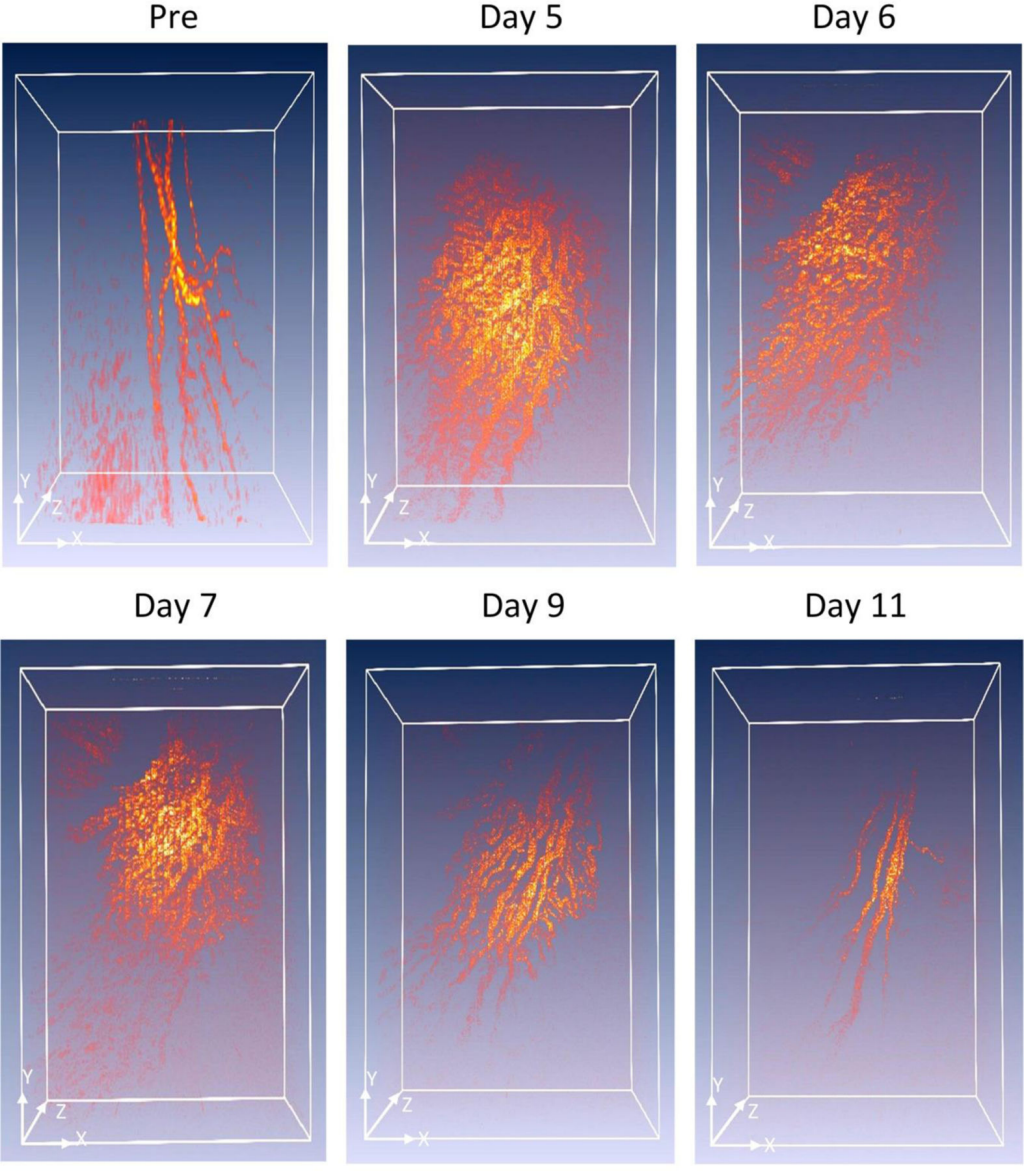
3D rendering PAM images.

**Figure 6:**
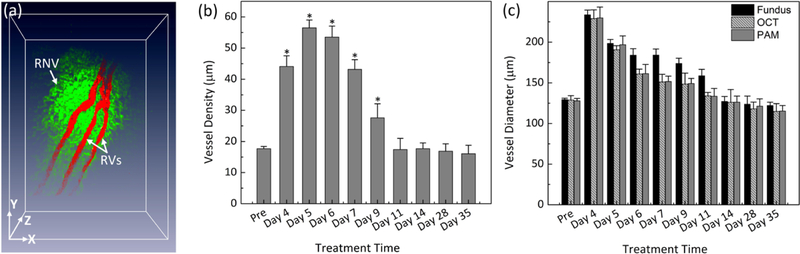
Quantitative analysis of retinal vessels after retinal neovascularizarion (RNV) model. (**a**) Segmentation image of retinal vessels for RNV identification. Pseudo-color red and green indicate the position of the existing retinal vessels and RNV, respectively. (b,c) Quantitative analysis of retinal vessels after RNV. (**b**) Vessel density as a function of treatment times (pre, day 4, 5, 6, 7, 9, 11, 14, 28, and day 35) (* p < 0.001 and N = 4). (**c**) Comparison vessel diameter measured from different methods: fundus, OCT and PAM (* p < 0.001 and N = 4).

To visualize the margin of the retinal vessel after VEGF injection and to examine the potential of OR-PAM for visualization of RNV, OR-PAM was performed in the rabbit models. The entire RNV was acquired and reconstructed in both 2D and 3D. Unlike FA, the OR- PAM image was obtained without injection of any exogenous contrast agents. Figure 4 shows the corresponding maximum intensity projection (MIP) OR-PAM image of the retinal neovascularization acquired along the selected scanning areas displayed in Figure A1. The OR-PAM image demonstrates the individual structure of the retinal vessels such as RNV was well as the choroidal vessels within the medullary ray with high contrast and high-resolution. The white arrows show the position of the major retinal vessels before injection whereas the blue arrows show the location of the RNV. The RNV vascular network matches well with the color fundus and FA images (Figure 2). The PAM image highlights the pathologic changes in the retinal vessels. The size and the vascular pattern of the major retinal vessels were evidently different and displayed increased vascular tortuosity. The retinal vessels after VEGF injection were dilated at days 4 and 5 before gradually reducing at days 6 and 7. In addition, the 3D volumetric rendering images of the vessels were reconstructed and illustrated in Figure 5. The 3D image was achieved from sequences of PAM B-scan cross-sectional images. The retinal microvascular network was better visualized and the entire margin of individual RNV, capillaries and choroidal vessels were visualized and distinguished, indicating the capacity of PAM for detection of individual blood vessels in vivo. Based upon the 3D image, those vessels were laid in different layers. Although Figure 5 illustrates a 3D reconstruction image of the retinal vessels with high image contrast, the extensive, closely knit branching network of retinal microvessels makes the detection of RNV quite difficult. Therefore, vessel segmentation algorithm was applied to precisely classify the margin of each retinal vessel and to trace the dynamic changes in vessel diameter. The boundary of the arteries, veins, RNVs, and choroidal vessels (CVs) was detected and distinctly segmented as displayed in Figure 6a. For classification purpose, all the retinal vessels were associated with different pseudo-colors (red, and green). The red color indicates the position of the existing retinal blood vessels whereas the green color identifies the formation of RNV. The entire structure and location of RNV are clearly identified, demonstrating the potential of PAM for detection and evaluation of RNV. In addition, the morphology of the major retinal vessels was also observed after segmentation. As shown in Figure 6a, the major retinal vessels were enlarged in comparison with one before VEGF injection. The in vivo PAM experiment demonstrated that PAM could serve as a potential technique to observe both the morphology of vasculature and neovascularization with high spatial resolution, high sensitivity and high specificity in single vessels. Acquired PAM volumetric data allowed the RNV to be observed precisely. In contrast, previous studies using FA to monitor RVO and could not visualize RVO in three dimensions [42,43].

To further examine the dynamic changes of the RNV model, vessel diameter and vessel density were quantified at various times after VEGF injection for a period of 5 weeks as shown in Figure 6b. The morphology and density of RNV was rapidly increased and achieved highest vessel density at day 5 to day 6 and then gradually decreased and became stable later on. The vessels density significantly decreased from day 9 to day 35. In fact, the vessels densities at day 9 and day 11 were 204% and 308% lower than that at day 5 (i.e., vessel density = 56.49 *±* 2.51%, 27.61 *±* 4.49%, and 17.38 *±* 3.61% for RNV at day 5, 9, and 11, respectively). It was noted that the vessel density was not significantly different from day 11 to day 35 (i.e., vessel density = 17.68 *±* 1.84%, 16.87 *±* 2.38%, and 16.05 *±* 2.74% for RNV at day 14, 28, and 35, respectively). The dynamic change of the major retinal vessels was also characterized (Figure 6c). The measurement was obtained from PAM, color fundus photographs, and OCT images. Overall, the diameter of the existing retinal vessels rapidly increased and reached peak at day 4. Apparently, the vessel diameter of the major retinal vessels before injection measured from PAM images exhibited 180% smaller than that of the major vessels after RNV model (vessel diameter = 127.86 *±* 3.02 µm for pre-injection vs. 229.80 *±* 13.46 µm for day 4 post-injection; *p* < 0.05), indicating that the retinal vessels pre-injection were thinner than the post-injection ones. Then, the estimated vessels were gradually decreased from day 5 to day 35. at day 14, 28 and day 35 (i.e., vessel diameter = 196.90 *±* 10.83 µm, 133.44 *±* 9.74 µm and 115.28 *±* 6.87 µm for days 5, 11, and 35 post-injection, respectively; *p* < 0.05). Similarly, the diameter of retinal vessels was also estimated to be 128.81 *±* 5.52 µm before injection and 229.03 *±* 10.79 µm for day 4 post-injection; (*p* < 0.01) from the OCT images. This is not significantly different from the results obtained from PAM images. From the fundus photography, the measured vessel diameter was also not significantly different. From fundus photography, the vessel diameter was measured to be 129.38 *±* 1.80 µm (pre-injection); *p* < 0.003, which was approximately 1.2% and 0.5% different from PAM and OCT measurements, respectively. Fundus photography consistently estimated slightly larger vessel sizes for both artery and veins at all times, whereas PAM and OCT were very similar. These results demonstrate that the size of the retinal vessel was dynamically changed compared to the vessels pre-injection.

**Figure 7:**
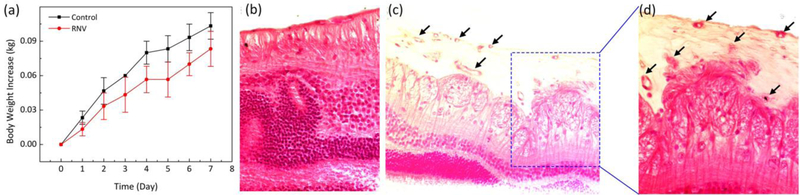
Histological analysis after intravitreal injection in rabbit retina. (**a**) Body weight increased as function of time after various treatment conditions. rabbits untreated with VEGF(control), rabbit groups treated with VEGF at dose of 100 L (10 g/mL). The body weight was obtained every day over a period of 7 days post-injection. The body weight was gradually increased for all treatment groups, and no difference was recognized between the treated and untreated groups, indicating that the VEGF at the applied doses are not toxic. Data expressed as mean SD (N = 4, p < 0.01). (**b**) H&E-stained images of retinal neovascularizarion (RNV) obtained from various groups: control (b), and treatment (c). The histological change and RNV development were observed at: (c) ×20 and (d) ×40.

To quantify any side effects and potential toxicity of VEGF application, the body weights of all treated animals were measured daily to reveal the overall condition of the rabbits [44]. In addition, chemistry panels consisting of creatinine (CREA), albumin (ALB), total protein (TPRO), calcium (CAL), alanine transaminase (ALT), blood urea nitrogen (BUN), and alkaline phosphate (AP) were analyzed to quantify the health of organs after administration of VEGF. A decrease in weight is commonly associated with treatment-induced toxicity in treated animals. Figure 7a shows that the body weight of the rabbits initially slight decreased on day 1 after treatment due to the prolonged anesthesia session and less oral intake that day. Then, the rabbit body weight gradually increased during the 7 days after different treatment conditions, suggesting that systemic toxicity was insignificant in the groups. The chemistry panel results show in Table 1. According to this result, ALP and BUN parameters are within the normal range, indicating that liver and kidney function are normal. Therefore, in vivo VEGF administration was correlated with minimal adverse effects and complications. To further evaluate the establishment of RNV, a standard histological staining with hematoxylin and eosin (H&E) was performed on the samples 35 days after injection (Figure 7b–d). As shown in Figure 7b–d, the morphology of cellular structures was not changed, and most nuclei were easily observed on the control histological images. Importantly, the tissue injected with VEGF showed that the morphology of tissue structures was changed, and new blood vessels were easily observed (Figure 7d). The black arrows depict the location of the newly developed blood vessels.

## 4. Discussion

The current study has successfully demonstrated a high-speed and non-invasive multimodal OR-PAM and SD-OCT imaging system with sufficient resolution for detection and visualization of retinal neovascularization in the rabbit retina. RNV was visualized and graded using dual-modality OR-PAM and SD-OCT without sacrificing the animal. This is important because OR-PAM is based on the optical absorption contrast of biological tissues and offers higher spatial resolution and three-dimensional capabilities to better characterize and assess the retinal vasculature at several time points. OR-PAM provides depth information in a non-invasive manner and is able to better assess the choroidal and deep vasculature better than SD-OCT. As a non-invasive imaging method, OCTA can generate angiographic imaging by employing motion contrast imaging to high-resolution volumetric blood flow information. Following the principle that movement in the back of the eye represents the blood flowed, the motion artifacts cannot be avoided in OCTA imaging. The minimum detectable flow velocity is determined by the time between the two sequential OCT B-scans, which causes it to miss some areas with slow blood flow. This is critical, as clinicians are most interested in slow blood flow, namely visualization of leakage from angiogenesis and microaneurysms. OCT and OCTA can provide high resolution structural and some functional information of the retina, but they have a limited ability to visualize the choroid and molecular imaging capabilities remain rudimentary. This is important because photoreceptors receive their blood supply from the choroid, a deeper vascular supply, which is particularly critical for the central vision, the fovea, where there is a foveal avascular zone (FAZ). OCT, OCTA, and other optical techniques have difficulty penetrating to this depth to visualize the choroidal vasculature with high resolution and instead report a choroidal thickness measurement, which serves as a poor biomarker for disease.

The current study combines SD-OCT and OR-PAM to overcome the limitations of SD-OCT and to better observe the retinal vascular network. In comparison with currently available imaging techniques, the multimodal system allows the detection and visualization of individual capillaries with high contrast and high resolution. FA imaging is widely used to evaluate the dynamic change of retinal neovascularization. Ishibazawa et al. and Edelman et al. have described that retinal neovascularization was able to be examined and graded using fluorescein angiography (FA) [45,46]. However, FA imaging could not determine the 3D depth of RNV, resulting in limitation of visualization of the blood vessel network. Liu, Jiao and Song et al. have developed an integrated PAM and OCT for visualization of normal and abnormal retinal vessels with promising results [17,22,26,47]. However, these studies could only be performed on small animals such as mice and rats. The eyes of mice are much smaller than human eyes (i.e., ~3 mm for mice, ~6 mm for rats vs. ~18mm for rabbits and ~23 mm for human), resulting in notable limitations of clinical applications. In contrast, the current study demonstrated that PAM visualized retinal neovascularization in living rabbits with high contrast to monitor the changes in microvascular structure and density over different time points. Importantly, PAM and OCT are able to detect and visualize the change of retinal vessels such as size and shape in both 2D and 3D. Optical resolution PAM allows one to evaluate and monitor the formation of RNV in 3D. In addition, high-resolution PAM systems could detect single microvessels without requiring exogenous contrast agents, which is extremely desirable for clinical applications. Another advantage of the current multimodal PAM and OCT is that the retinal vessels can be rapidly monitored with OCT and PAM system to achieve high-resolution images in less than one minute. A study reported by Robinson et al. shows that the eye has a very short fixation time of approximately 500 ms [48]. The motion artifacts can cause image blurring or image disruption. The multimodal imaging system has excellent lateral and axial resolution of 4.1 and 37 µm for PAM and 3.8 and 3.0 µm for OCT which is suitable for detection of individual capillaries with the diameter less than 10 µm. However, the current PAM system could not monitor the retinal vessels in real time due to the limitation in acquisition time. In comparison to the acquisition time of FA and color fundus photography (~1 s), the acquisition times of PAM and OCT are approximately 60 and 10 times longer. The acquisition time is limited by the laser repetition rate of 1 kHz of the OPO for PAM, and 36 kHz for OCT. as shown in Table 2. However, the acquisition time can be improved by increasing the laser repetition rate. For example, a reported by Liu and Song et al. described that the acquisition time is about 2.7 s to achieve a raster-scan region of 2 *×* 2 mm using optical scanning method [22,26,49].

While PAM can provide excellent image contrast and reasonable resolution, several extra safety evaluations should be further performed prior to translate this imaging technique to clinical applications. Jiao et al. has applied laser energy of 40 nJ to achieve PAM of the iris in mice, Kelly Goss and Joen et al. have used the illumination light of 50 nJ and 120 nJ, respectively to detect corneal neovascularization in mice model [16,22,37]. Chao et al. applied the laser energy of 80 nJ for detection of normal chorioretinal vessels in living rabbits [29]. The current performed PAM to distinguish individual retinal neovascularization at low laser exposure energy of 80 nJ at 580 nm, a half of the ANSI safety limit, long-term effect should be examined and monitored. Although near-infrared (NIR) laser light (780–1200 nm) could provide deeper imaging than green and blue laser light due to low level of hemoglobin absorption [27,50,51], it requires applying a higher illumination energy to achieve a high-contrast image [33]. In contrast, hemoglobin strongly absorbs visible light. Thus, the exposure dose could be reduced, and a high contrast image could be achieved within the ANSI safety limit for the eye. In addition, the most important requirement for PAM imaging of the eye is to rapidly achieve high contrast and high-resolution images without affecting or damaging sensitive neural tissue. Although the current study applied laser dose below ANSI safety limit, long-term thermal damage, thermoacoustic damage, and photochemical damage can impact the retina [52,53]. Thus, further safety evaluations will be performed to evaluate the potential long-term effect of illumination light to the eye before translating this imaging technique to the clinic.

This study also demonstrated that injection of VEGF within the rabbit vitreous induces retinal neovascularization in the rabbit eyes. The histopathologic evidence of RNV was established in 100% of lesions, which is consistent with previously report by Wong et al. [54]. Please note that the existing retinal vessels were dilated after injection due to the early stages of inflammation in the rabbit’s retina after injection. The VEGF-induced RNV model showed that mild vascular change without progression to vitreous hemorrhage or retinal detachment. In addition, no signs of dilation of conjunctival blood vessels were observed. The retinal neovascularization is produced rapidly in the rabbit by intravitreal injection of VEGF within 3–4 days. However, one limitation of the RNV model-induced by intravitreal implantation of VEGF is that the new blood vessels dynamically changed as a function of time as shown in Figure 2, and Figure 4. The progression of RNV was monitored using OR-PAM. The acquired MIP PAM images (Figure 4) showed that the RNV vessels were obviously identified with high contrast. The morphology of the RNV network such as size, shape, and density can be quantitatively performed on different images, including color fundus photography, SD-OCT and OR-PAM for quantification of the developed vessels as a function of time. Moreover, the highly reproducible development of these vascular changes in this rabbit model with a short time frame suggests that potential therapeutic modalities for intervention of angiogenesis can be tested readily. Importantly, advantages of the RNV model in rabbit include the close approximation to human eyeball axial length, which is an important step for studies of RNV with PAM and utility of the RNV model for development of human clinical trials.

## 5. Conclusions

In summary, this study demonstrates that retinal neovascularization can be observed and evaluated using multimodal SD-OCT and OR-PAM in vivo in rabbits. Experimental results demonstrated that the multimodal SD-OCT and OR-PAM imaging system have a high potential for detection of individual microvessels with high resolution and high contrast. The OR-PAM modality could non-invasively, non-ionizing, and label free distinguish individual retinal neovascularization at low laser exposure energy of 80 nJ, which is half of the ANSI safety limit. The OCT system could help to quantify single RNV capillaries and distinguish different layers of RNV, choroid, and sclera. In addition, both PAM and OCT can visualize the change in retinal thickness. Therefore, the multimodal ocular imaging system can be effective and safe for visualization and characterization of the retinal neovascularization diseases.

## Figures and Tables

**Table 1.** Mini chemistry test results.

Test	Unit	Reference Range	Control	VEGF
CREA	mg/dL	0.5–2.6	1.21	1.08
ALB	g/dL	2.7–5	4.3	4.1
TPRO	g/dL	5–7.5	6.6	5.8
CALA	mg/dL	5.6–12.1	12.4	13.6
ALT	U/L	25–65	44	32
BUN	mg/dL	5–25	18	19
AP	U/L	10–86	83	85

**Table 2.** Comparison of ocular imaging modalities.

Specifications	Color Fundus	FA	SD-OCT	PAM
Axial Resolution			4.0 µm	37.0 µm
Lateral Resolution			3.8 µm	4.1 µm
Contrast Mechanism	Reflected illumination light from a flash bulb	Exogenous contrast agents	Back-scattering photons from chromophores and hemoglobin	Absorption deposited laser energy of hemoglobin

Acquisition Time:				
- B-scan Image	No	No	0.02 s	0.2 s
- 2D Image	1 s	1 s	9.7 s	60 s
Imaging Depth	No	No	~1.8 mm	~1–3 mm
Retinal Vessels	Yes	Yes	Yes	Yes
Choroidal Vessels	Yes	Yes	Yes	Yes
Retinal Neovascularization	Yes	Yes	Yes	Yes
Capillaries	Yes	Yes	Yes	Yes
Field of view (FOV)	40°	40°	8 × 8 mm	4 × 4 mm
3D volumetric Image	No	No	Yes	Yes

## Highlights

1. The characterization of intravitreal VEGF injection-induced retinal neovascularization was evaluated by a custom-built multimodal PAM and OCT imaging system.
2. Acquired PAM imaging provides 3D volumetric images of the vascular network that allowed us to observe the margin of retinal neovascularization.
3. Integrated PAM and OCT imaging allowed detection of individual retinal neovascularization and to observe the changes in microvasculature morphology post VEGF injection.
